# The Effects of Various Teaching Methods on Chest X-ray Interpretation Skills Among Medical Students and Interns: A Systematic Review

**DOI:** 10.7759/cureus.44399

**Published:** 2023-08-30

**Authors:** Abdulmohsen Al Elq, Abdulaziz A Alfayez, Muhannad I AlQahtani, Rakan S Alshahrani, Ghazi A Alotaibi, Abdullah A Aldakheel, Ali A Alhammad, Radwa H Bakr Mohamed, Mu’taman Jarrar, Mai Abdel Haleem A Abusalah, Mohammad Al-Bsheish

**Affiliations:** 1 Department of Internal Medicine, College of Medicine, Imam Abdulrahman Bin Faisal University, Dammam, SAU; 2 College of Medicine, Imam Abdulrahman Bin Faisal University, Dammam, SAU; 3 Vice Deanship for Development and Community Partnership, College of Medicine, Imam Abdulrahman Bin Faisal University, Dammam, SAU; 4 Department of Medical Education, King Fahd Hospital of the University, Al-Khobar, SAU; 5 Department of Medical Laboratory Sciences, Faculty of Allied Medical Sciences, Zarqa University, Al-Zarqa, JOR; 6 Department of Health Management, Batterjee Medical College, Jeddah, SAU; 7 Department of Occupational Health, Al-Nadeem Governmental Hospital, Ministry of Health, Amman, JOR

**Keywords:** chest x-ray, rayyan platform, pico framework, systematic review, chest x-ray interpretation, medical students

## Abstract

Chest X-ray (CXR) is a common tool used in medical practice. Medical students and interns should acquire knowledge of CXR interpretation, as it is an essential diagnostic tool for a large spectrum of diseases.

This systematic review aimed to compare the effect of different intervention techniques on the competency of medical students and interns to demonstrate the level of confidence and competence in interpreting common presentations of CXRs.

The population, intervention, comparison, and outcomes (PICO) framework was used to formulate the review question. All related articles in five databases (PubMed, Web of Science, Scopus, Medline, and Embase) were retrieved and the search was completed in March 2023 with no limiters on date and time. The number of relevant studies was 469. A multi-level approach through the Rayyan platform was used for the screening and exclusion processes.

Eleven articles were included in the systematic review consisting of eight randomized controlled trials, one quasi-experimental study, one cross-sectional study, and one interventional cohort. Results showed significant effects of teaching methods utilizing deductive or inductive approach, clinical history, patient care comfort survey, and SAFMEDS (Say-All-Fast-Minute-Every-Day-Shuffled). Contrarily, no significant effect was shown by flipped classroom models and mixed and blocked practice, peer-assisted learning vs. expert-assisted learning, and Chester, an artificial intelligence tool.

This review identified beneficial approaches that may enhance the learning outcomes of interpreting CXRs for medical students and interns, highlighting the remarkable impact of SAFMEDS on medical students' ability to identify CXR findings as well as the availability and practicality of online and e-learning resources for students.

## Introduction and background

Chest X-ray (CXR) is a common diagnostic tool used in medical practice that is a fast radiological imaging modality enabling the assessment and evaluation of internal chest structures and organs, including the heart, lungs, airways, bones, and pleural cavities [1,2]. Furthermore, the most common imaging modality being used worldwide is CXR, and even though it is cognitively demanding and requires experience, it is taught less frequently today [3-6]. In addition, CXR continues to have a crucial role in diagnosing and detecting several disorders and non-chest-related conditions, such as bowel perforation and preoperative assessments of patients, despite modern medical advancements [7-9].

Future medical doctors are expected to successfully identify common radiological findings/pathologies because clinical decisions based on incorrect interpretations may have adverse consequences for patient care as the radiologist's report impacts patient management [10]. Hence, some medical schools introduce CXR to medical students as early as their first year [11,12]; however, there is a lack of formal, comprehensive, and advanced training provided throughout the clinical practice year. Moreover, only 5% of total teaching time in medical schools is dedicated to radiographic examination tools [11,13]. As a result, undergraduate and postgraduate trainees feel a lack of preparedness for clinical practice in the field of radiology related to a mismatch between the received radiology teaching and their learning needs [14,15].

Due to the potential repercussions of incorrect CXR interpretation, accurate interpretation is essential for investigating and managing diseases. Despite CXR’s significant role in saving lives, it can endanger lives if interpreted improperly or with inadequate knowledge [7,10]. Therefore, early training and education in basic diagnostic imaging are recommended [14].

It is vital to analyze medical students' ability for CXR interpretation during their clinical years to determine their preparedness for future careers. There is a paucity of literature regarding medical students' ability to interpret CXR and the most appropriate teaching methods for enhancing medical students' and interns’ radiological competencies. Therefore, this systematic review was conducted to study medical students' and interns' competency in interpreting CXR and to explore the most appropriate teaching method that may contribute to curriculum development.

## Review

Methods

The population, intervention, comparison, and outcomes (PICO) framework, was used to formulate the review question: “How do different methods of interventions impact the competency of medical students and interns in interpreting CXR?” The criteria defined in the Preferred Reporting Items for Systematic Reviews and Meta-Analyses (PRISMA) guidelines for systematic review and meta-analysis were applied in this review [16].

Search Strategy

**Figure 1:**
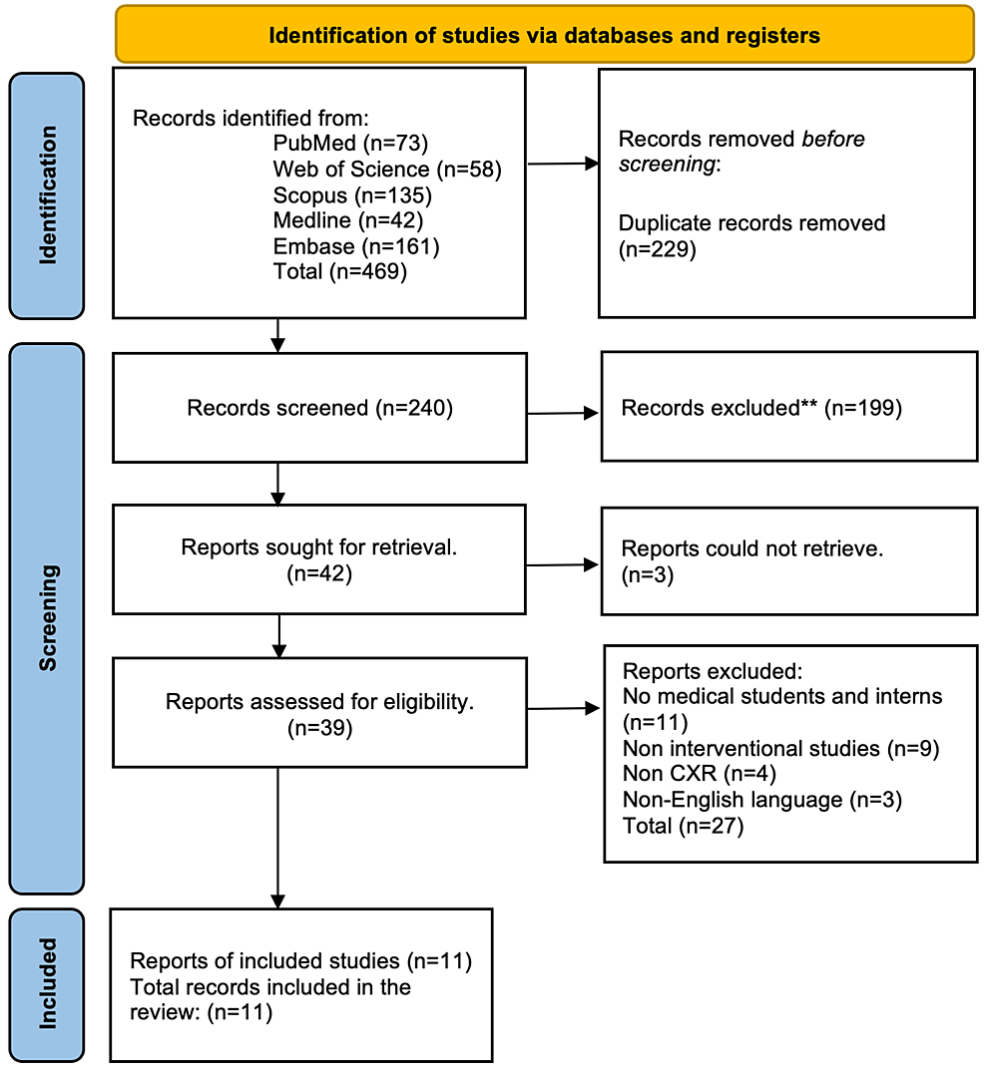
Flowchart of the literature search selection and screening strategy ** Non-medical students, animal studies, non-chest X-rays, e.g., CT, MRI, and ultrasound, non-educational, and studies that do not measure competency.

A comprehensive search was done using PubMed, Web of Science, Scopus, Medline, and Embase. Databases were searched from 1968 until March 2023, with the limitation of solely selecting publications in English. The relevant Medical Subject Headings (MeSH) terms and keywords were used to ensure that all relevant inclusion criteria items were identified. The Boolean operators "AND/OR" were applied between search terms. The keywords utilized in this systematic review were ("chest X ray" or "chest X-ray" or "CXR") AND ("interpretation" or "reading" or "evaluation”) AND ("medical students" or "students" or "medical education"). All the article bibliographies obtained were examined for further relevant publications. Two reviewers independently examined the titles and abstracts of all potentially relevant studies. All relevant studies were imported into and examined for duplicates using EndNote version X8 (Clarivate, London, UK). Any disagreements between the two reviewers were discussed and resolved effectively after consulting a third reviewer. Figure 1 demonstrates the flowchart of the literature search selection and screening strategy.

Eligibility Criteria

This review included studies based on several criteria. First, the primary study type was interventional studies. Second, the included studies targeted medical students, interns, or junior doctors as their population sample. Finally, this review included studies that used CXR as an imaging modality to evaluate the students' competency measured as an outcome, and articles published in English. Studies wherein the modality was other than CXR, e.g., CT, MRI, and US, or the language, was not English, were excluded. The search was completed in March 2023 with no limiters on date and time.

Data Extraction

Rayyan software [17], a web-based platform that enables reviewers to cooperate throughout the screening process, was used. All included studies were selected based on the titles and abstracts by a panel of three researchers (Alshahrani R, Alotaibi G, and Alqahtani M). Full articles were obtained from the databases if they met the inclusion criteria of the systematic review. In addition, the papers that were initially excluded were re-examined to ensure that no relevant articles were missed in the first screening stage. Specific items required for data extraction were gathered for each included study.

Study Quality Assessment

The Joanna Briggs Institute (JBI) critical appraisal tools for systematic review were used to assess the methodological quality of the included studies and to determine the extent to which a study has addressed the possibility of bias in its design, conduct, and analysis [18]. Each JBI appraisal tool consists of multiple questions with four possible answers: yes, no, unclear, and not applicable.

Results

Study Selection

A total of 469 studies were obtained by online database searches. After the removal of duplicated studies, 240 articles were screened. During the screening, 199 studies deemed ineligible based on language and article type were excluded. The 42 remaining studies were then carefully evaluated by examining each study's abstract and full text, of which three were deemed ineligible. Following that, 35 articles were further evaluated, and 27 were removed as they did not fulfill the inclusion criteria of our systematic review. Finally, the comprehensive screening identified 11 studies that met the inclusion criteria. The 11 included studies were published between 2012 and 2022 in this systematic review.

Characteristics of the Included Studies

Table 1 summarizes the main characteristics of the included studies [9,19-29]. The included studies originated from different parts of the world with various interventional study designs that included randomized controlled trials (RCTs) (n = 8) with a sample size between 14 [19] and 166 [20], quasi-experimental study (n = 1) with 40 medical students [21], cross-sectional study (n = 1) with 99 non-radiologists of whom 38 were medical students [9], interventional cohort (n = 1) with 152 first-year graduate entry [22], and other interventional studies (n = 1) with 18 medical students [23]. Studies took place in the Netherlands, Ghana, Pakistan, the United States of America, Ireland, Canada, Saudi Arabia, and Australia.

**Table 1:** Characteristics of the included studies * SAFMEDS: Say-All-Fast-Minute-Every-Day-Shuffled. ** Normal radiograph, bronchiectasis, miliary disease, pneumothorax, lung mass, emphysema, pleural effusion, pneumonia, atelectasis, congestive heart failure, pulmonary fibrosis, and mediastinal mass.

1. Author(s)	2. Publication year	3. Sample	4. Setting	5. Instruments used or intervention (i.e., questionnaire or 4-day workshop: presentation teaching or simulation)	6. Study design	7. Measures (i.e., Pre-test and post-test questionnaire or Pre-test and 2-week post-test questionnaire)
1. Geel et al. [23]	2019	Third-year medical students (n = 103)	Maastricht University in the Netherlands	A 2 x 2 design of the proportion of normal images and a deductive or inductive approach	Randomized controlled trial	Post-test of 20 chest radiographs, of which 60% were normal images
2. Jimah et al. [9]	2020	99 non-radiologists comprising 10 residents, 18 medical officers, 33 house officers, and 38 final-year medical students	Cape Coast Teaching Hospital (CCTH) in the Central Region, Ghana	A semi-structured questionnaire with and without clinical history	Cross-sectional study	10 radiograph questionnaires in two phases: first without clinical history, and then with a clinical history of the same cases in the same sequence
3. Afzal and Masroor [21]	2019	40 third-year students	Radiology Department, The Aga Khan University Hospital, Karachi	Flipped classroom model	Quasi-experimental study	Assessment by radiology case scenarios at the end. A simplified survey and two open-ended questions for feedback
4. Shelesky et al. [19]	2012	14 medical interns	UPMC St Margaret Hospital, Pittsburgh, Pennsylvania	The treatment group received direct observation (DO) with formal feedback (FF) by a senior resident or faculty member	Randomized controlled trial	Administration of patient care comfort survey (PCCS) at baseline, 6 weeks, and 12 weeks. Additional videotaping occurred periodically
5. Dunne et al. [27]	2022	40 third-year medical students: intervention group (n = 20) and control group (n = 20)	The National University of Ireland, Galway City, Ireland	SAFMEDS*	Randomized controlled trial	Baseline, post-test, and retention tests
6. Shah et al. [26]	2016	58 medical students from 1-3 years	The University of Toronto	Blocked and mixed modules	Randomized controlled trial	Immediate post-test after completing the modules, then after two weeks
7. Dao et al. [29]	2022	32 third-year medical student	University of Montreal	Chester	Randomized controlled trial	Pre-post intervention survey and two test exams
8. Rozenshtein et al. [24]	2016	40 medical students (24 first-year medical students and 16 second-year students)	Columbia University	A presentation consisting of six examples of 12 common radiographic patterns**	Randomized controlled trial	A multiple-choice test (consisting of two cases in each radiographic pattern, one previously shown and one new) was given after a distraction break
9. Wong et al. [25]	2015	99 medical students (from years 1-4 of the 6-year program)	The University of New South Wales, Sydney, Australia	Adaptive tutorial and peer-reviewed web resource (Diagnostic Imaging Pathways website). Both were of one-week duration	Randomized controlled trial	Timed examination-style online assessment. The User Engagement Scale (UES) questionnaire assessed students' engagement with the learning resource
10. Alsulmi et al. [20]	2022	166 second-year medical students	Umm Al-Qura University, Makkah, Saudi Arabia	Peer-assisted learning (PAL) and expert-assisted learning (EAL)	Randomized controlled trial	Online form for demographics and consent. The online form of a test and the modified Mills et al. survey. A post-online test and survey
11. Wentzell et al. [28]	2018	First-year graduate entry (n = 152)	The University of Limerick, Ireland	An online web-based intervention	Cohort study	1. A pre-, mid-, and post-assessment. 2. A confidence and feedback questionnaire

The tools for the intervention ranged from post-test only in Geel et al. [23], Rozenshtein et al. [24], and Wong et al. [25] to assess a 2 x 2 design testing the effects of the proportion of normal images and deductive versus the inductive approach, massed versus interleaved teaching method, and adaptive tutorial versus peer-reviewed web resource, respectively. In addition, a User Engagement Scale (UES) was used to measure student engagement with the online resource for Wong et al.'s [25] study, in which students rated adaptive tutorial eight out of 10 in comparison to six for the Diagnostic Imaging Pathways' website [25]. The same applied to Shah et al. [26], with the addition of another test after two weeks for measuring retention, while Dunne et al. [27] used the retention test with pre- and post-tests to assess the SAFMED (Say-All-Fast-Minute-Every-Day-Shuffled) method of intervention.

Moreover, Afzal and Masroor [21] used only surveys as feedback for the flipped classroom module at the end of the intervention. In Shelesky et al.'s study [19], interns completed the patient care comfort survey (PCCS), a tool that measures the intern’s comfort levels in history taking, examination, investigation, and diagnosis. PCCS was measured at the beginning of the study, at six and 12-week intervals, in addition to videotaping occurring for all interns while on-call at approximately the beginning, middle, and end of the study period to assess competency and confidence after having the direct observation (DO) with formal feedback (FF) four times per month on different skills, including CXR interpretation.

On the other hand, a mix of measures of assessment was used to assess the effect of an online web-based blocked practice focusing solely on cardiac interpretation with step-by-step feedback, which McMaster University developed in a study by Wentzell et al. [28] where pre, mid, and post assessments were used, followed by a confidence level survey.

Furthermore, the study by Alsulmi et al. [20] assessed the intervention of peer-assisted learning (PAL) vs. expert-assisted learning (EAL) through a mid-online test and a post-online test, which both included a satisfaction survey. In addition, Dao et al. [29] used pre- and post-intervention surveys with two tests: exam 1, with the aid of Chester, an artificial intelligence (AI) tool, for the experimental group, and exam 2, without using the tool for both the experimental and control groups. Further, Jimah et al. [9] administered two tests, a pre-test without clinical history and a post-test including clinical history, to assess the accuracy and competency of CXR interpretation concerning clinical history.

Some studies exhibited limited benefits in their method of intervention. For example, Afzal and Masroor [21] showed no significant impact of the flipped classroom model measured by the end-of-clerkship students’ grades. However, there was an increased satisfaction and perceived value of this model by the students. Shah et al. [26] also exhibited no obvious disparities in mixed and blocked practice outcomes. Dao et al. [29] found no statistically significant impact on the experimental group’s performance or group’s performance or confidence level when compared to the control group. Similarly, Alsulmi et al. [20] found no significant discrepancies between the two groups, but the PAL group had higher satisfaction.

Additionally, Geel et al.'s [23] study demonstrated that the deductive method shown was better in results than the inductive approach in judging normal vs. abnormal CXR and that the criteria by which students categorized normal and abnormal CXR were affected by the ratio of normal to abnormal CXR. Although no pre-test was used to measure the retention of knowledge, the students were described as novices who had acquired knowledge of chest pathology during pathology training, which could have affected the results in the deductive approach. Moreover, Jimah et al. [9] demonstrated that clinical history greatly impacted the competency of doctors and medical students and that a gap existed in the competency between decision-making and the cooperation of the patients. Further, Shelesky et al. [19] showed that for certain areas, comfort with patient care skills (CWPCS) increased in the treatment group compared to the control group. There was a lack of perceived comfort at six weeks by the control group in each of the eight significant questions on the PCCS. Dunne et al. [27] found that a considerable effect on the intervention was noticed with SAFMEDS, which indicated its significant effectiveness. Furthermore, in Rozenshtein et al.'s [24] study, the interleaved (spaced) instruction method showed better outcomes than the massed method across all education levels. As the study’s results suggested, spacing is helpful in recollection and may also be helpful in the induction of pattern recognition in radiology. Wong et al. [25] found that higher assessment scores were noticed in the adaptive tutorials group compared to the control group. Students showed better engagement with adaptive tutorials than web resources and evaluated the tutorials as a remarkably useful learning instrument. Specifically, adaptive tutorials on diagnostic imaging showed notable quantitative and qualitative learning advantages for senior medical students. Also, Wentzell et al. [28] found an improvement in the interpretation skill (basic) and confidence level and a stronger correlation between the diagnosis assessment score and the diagnosis confidence.

Study Quality Assessment

A critical appraisal of all 11 studies included in this systematic review was conducted via the aforementioned JBI appraisal tools. Tables 2-5 summarize the results of the individual performance of each study. Most included studies showed a high percentage of positive answers to the questions of the JBI tool.

**Table 2:** Representation of studies performance for RCTs RCTs: randomized controlled trials; JBI: Joanna Briggs Institute.

Representation of individual studies performance according to the JBI critical appraisal tool for RCTs														
Author name/year	Was true randomization used for the assignment of participants to treatment groups?	Was allocation to treatment groups concealed?	Were treatment groups similar at the baseline?	Were participants blind to treatment assignment?	Were those delivering treatment blind to treatment assignment?	Were outcome assessors blind to treatment assignment?	Were treatment groups treated identically other than the intervention of interest?	Was follow-up complete, and if not, were differences between groups in terms of their follow-up adequately described and analyzed?	Were participants analyzed in the groups to which they were randomized?	Were outcomes measured in the same way for treatment groups?	Were outcomes measured reliably?	Was appropriate statistical analysis used?	Was the trial design appropriate, and were any deviations from the standard RCT design (individual randomization, parallel groups) accounted for in the conduct and analysis of the trial?	Total score (%)
Geel et al. (2019) [23]	Yes	Unclear	Yes	Yes	No	Unclear	Yes	Yes	No	Yes	Yes	Yes	Yes	11/13 (84.6)
Shelesky et al. (2012) [19]	Yes	Yes	Yes	No	No	Yes	Yes	Yes	Yes	Yes	Yes	Yes	Yes	11/13 (84.6)
Dunne et al. (2022) [27]	Yes	No	Yes	No	No	No	NA	Yes	Yes	Yes	Yes	Yes	Yes	8/13 (61.5)
Shah et al. (2016) [26]	Yes	NA	Yes	Unclear	NA	Yes	NA	Yes	Yes	Yes	Yes	Yes	Yes	9/13 (69.2)
Dao et al. (2022) [29]	Yes	Yes	Unclear	No	NA	NA	Yes	Yes	Yes	Yes	Yes	Yes	Yes	10/13 (76.9)
Rozenshtein et al. (2016) [24]	Yes	Yes	No	Yes	Yes	No	Yes	Yes	Yes	Yes	Yes	Yes	Yes	11/13 (84.6)
Wong et al. (2015) [25]	Yes	Yes	Yes	Yes	Yes	No	Yes	Yes	Yes	Yes	Yes	Yes	Yes	12/13 (92.3)
Alsulmi et al. (2022) [20]	Yes	Yes	Yes	Yes	NA	No	Yes	Yes	Yes	Yes	Yes	Yes	Yes	11/13 (84.6)

**Table 3:** Representation of studies performance for cohort studies JBI: Joanna Briggs Institute.

Representation of individual studies performance according to the JBI critical appraisal tool for cohort studies												
Author name/year	Were the two groups similar and recruited from the same population?	Were the exposures measured similarly to assign people to exposed and unexposed groups?	Was the exposure measured validly and reliably?	Were confounding factors identified?	Were strategies to deal with confounding factors stated?	Were the groups/participants free of the outcome at the start of the study (or at the moment of exposure)?	Were the outcomes measured validly and reliably?	Was the follow-up time reported and sufficient to be long enough for outcomes to occur?	Was follow-up complete, and if not, were the reasons for loss to follow-up described and explored?	Were strategies to address incomplete follow-up utilized?	Was appropriate statistical analysis used?	Total score (%)
Wentzell et al. (2018) [28]	NA	No	Yes	No	No	No	Yes	Yes	Yes	No	Yes	5/11 (45.5)

**Table 4:** Representation of studies performance for cross-sectional studies JBI: Joanna Briggs Institute.

Representation of individual studies performance according to the JBI critical appraisal tool for cross-sectional studies									
Author name/year	Were the criteria for inclusion in the sample clearly defined?	Were the study subjects and the setting described in detail?	Was the exposure measured validly and reliably?	Were objective, standard criteria used for the measurement of the condition?	Were confounding factors identified?	Were strategies to deal with confounding factors stated?	Were the outcomes measured validly and reliably?	Was appropriate statistical analysis used?	Total score (%)
Jimah et al. (2020) [9]	No	Yes	Yes	NA	No	No	Yes	Yes	4/8 (50.0)

**Table 5:** Representation of studies performance for quasi-experimental studies JBI: Joanna Briggs Institute.

Representation of individual studies performance according to the JBI critical appraisal tool for quasi-experimental studies										
Author name/year	Is it clear in the study what is the "cause" and what is the "effect" (i.e., there is no confusion about which variable comes first)?	Were the participants included in any comparisons similar?	Were the participants included in any comparisons receiving similar treatment/care other than the exposure or intervention of interest?	Was there a control group?	Were there multiple outcome measurements, both pre and post-intervention/exposure?	Was follow-up complete, and if not, were differences between groups in terms of their follow-up adequately described and analyzed?	Were the outcomes of participants included in any comparisons measured in the same way?	Were outcomes measured reliably?	Was appropriate statistical analysis used?	Total score (%)
Afzal and Masroor (2019) [21]	Yes	Yes	Yes	Yes	No	NA	Yes	Yes	Yes	7/9 (77.8)

Discussion

In this systematic review, we screened 240 studies, of which a few targeted medical students and interns, and measured their competency and confidence levels. For instance, the national cross-sectional study by Samara et al. [30] revealed a high confidence level among senior medical students and yet showed a variety of scoring between different universities due to the variations in curricula. Additionally, a comparative study by Eisen et al. [22] found that although interpretation improved with training, important diagnoses were missed and recommended more effective training, especially in radiographic emergencies. Similarly, Cheung et al. [31] highlighted the importance of structured radiology methods of teaching that should aim at the early postgraduate year levels and recommended further research of the factors influencing confidence that could result in improvement in CXR interpretation. Accordingly, this study supported these recommendations after a detailed inspection of 11 interventional studies that aimed toward finding better methods to enhance the competencies of medical students and interns in interpreting CXR.

Different variables were involved in synthesizing these studies aiming to improve medical students’ and interns’ performance, possibly influencing the results. Afzal and Masroor [21] found that the flipped classroom model met with a positive response from students undertaking private sessions, which correlates with a similar response in Park et al. [32] and Alsulmi et al. [20]. Nonetheless, when looking into the data in these studies, there was no impact or significant differences in a student’s performance or overall evaluation. Such positive reactions from students to the interactive session might be due to the students’ being more comfortable in face-to-face sessions [21,32] or in the online setting [20], where the students were on time and felt a better understanding of their lessons on the PAL. Likewise, in Dao et al.'s [29] study, neither students’ diagnostic performance nor confidence level improved with Chester. However, 90% of students gave a positive impression regarding the usefulness of Chester. Because it was a pilot study with a small sample size, the statistical strength and reliability of the study might be affected. Second, Chester had inadequate diagnostic accuracy in exam 1, even if the questions were deemed appropriate for third-year medical students. On top of that, Chester has a labeling bias as it is programmed on labeled images and analyzes only frontal X-ray images, which might affect a student’s performance and educational process. Different AI tools could be utilized with the study’s methodology as it is robust, and more developed AI tools could yield better results with more funding and widespread utilization.

Another tested variable was the influence of clinical history on performance, which was evident in Jimah et al.'s [9] study. The study found that clinical history greatly impacted the competency of doctors and medical students, thus recommending and advising that a correct and sufficient patient history should be provided when asking for radiography. Also, focusing on the radiology course during medical training is needed to enhance the confidence of doctors and their competence [9,22,33,34].

Compared with earlier tested techniques, such as the training module, SAFMEDS showed a better effect on the performance of CXR interpretation, although it is less decisive regarding the comparison of fluent and non-fluent participants, as Dunne et al. [27] stated. However, SAFMEDS focuses not only on respiratory conditions but also on multiple additional abnormalities. Further, it could be used at any time. Also, the online web-based intervention by McMaster University that was used as a blocked practice for students concentrating solely on cardiac interpretation in Wentzell et al. [28] showed improved basic CXR interpretation relating to the heart and confidence level. However, the overall performance was poor due to the brief study period and the little to no degree of knowledge the students had, considering they were starting medical school and that this was their first exposure to medicine. Accordingly, the authors suggested that skill and confidence would increase with more exposure and training, which students would attain as they progressed to clinical years. However, the study lacked a subjective analysis of confidence level changes over time, and it used cases similar to the pre-mid-post intervention, which could have created some bias.

In Rozenshtein et al.'s [24] study, the difference between interleaved and massed teaching approaches was examined by a multiple-choice test that included cases related to each radiographic pattern (two cases for each pattern; one of which had been shown before and the other one was new). The small sample size, testing the immediate effect rather than the long-term memory retention required in real-life practice, and the impossibility of the results to be applied on radiology residents, fellows, or any higher-level trainees were considered limitations of this study. Besides, the study lacked an evaluation of a student’s satisfaction regarding both approaches, and a student’s prior knowledge of the number of cases for each pattern could have led to bias. Further research was recommended for several reasons, including covering a higher number of students in the sample, evaluating which approach the students prefer, and assessing the effect of spacing on recollection and induction after a period of time. Also, before asserting the effectiveness of the interleaved approach, research on higher-level trainees should be done.

Furthermore, Wong et al. [25] assessed the effect of adaptive tutorial on interpreting and using CXR and CT for the head, chest, and abdomen by comparing it to the Diagnostic Imaging Pathways website, a peer-reviewed web resource. The comparison was done via an online assessment in a timed examination style. The sample size was small to some extent, and the researchers provided minimum clinical data with questions that needed interpretation. Two possible sources of bias in this study included selection bias, as the participants might be interested in diagnostic imaging more than their classmates, and the fact that there were no incentives for the students regarding their course credit. Cronbach’s α values of 0.6 and 0.62 resulted in chest X-ray and CT scan examination-style assessments, respectively, which corresponds to less-than-ideal reliability, yet values were still acceptable. Since it is a crossover study, and there is a possible inaccuracy in students' estimation of the time they spent studying the educational resources, there might be a risk of carryover effects between the CXR and CT parts of the study. The researchers suggested further studies on a larger sample size and new medical graduates after experiencing the use of adaptive tutorials by the Biomedical Education Skills and Training (BEST) network. As mentioned previously, the assessment of students' engagement with the sources was done via the UES questionnaire.

Shah et al. [26] hypothesized that a mixed module rather than a block module would be superior in students’ teaching and performance. However, the study results did not match the study hypothesis, as the mixed practice module did not show any advantage in the CXR interpretation, and both results were similar. Evidence indicated that block modules hinted about the concept in the question as the learner had just finished it. However, in the mixed module, learners needed more cognitive functions to confirm the concept before answering test questions. This study is distinctive as it had multiple strengths, including the flexibility to select the participants’ learning time, the randomization technique used, and the reduced instructors’ confounding effects on the learning process as they were self-study modules. This study might not be completely appropriate compared to others in the field of mixed and blocked practice modules, as it contained a visual task while other studies included different types of skills, such as language skills.

While reviewing these studies, a few limitations and possible biases were noted in their methodology or implementation phase. In the study conducted by Jimah et al. [9], participants in the second phase of the study were given the same test in the same sequence with a difference in clinical history, which can create bias in answering the questions. Therefore, it was recommended to change the images with the same pathology similar to Wentzell et al. [28]. Additionally, the study by Jimah et al. [9] had a small sample size and was carried out in a single institution (the Cape Coast Teaching Hospital). Another limitation was that Shah et al. [26] intentionally avoided using baseline performance by pre-test evaluation to prevent test-enhanced learning. Such evaluation is important in providing a valuable indication of the intervention's effectiveness.

Moreover, there was a possibility in Shah et al.'s [26] study for participants to use assistance from external sources. Further, there was a lack of clinical presentation and parallel teaching in Dunne et al.'s [27] study, which may have limited the accuracy of the results. Similar to Shah et al., studies by Geel et al. [23], Rozenshtein et al. [24], Wong et al. [25], and Alsulmi et al. [20] also did not have a pre-test, which is considered a limitation of their studies.

Shelesky et al. [19] showed that the treatment group’s CWPCS increased compared to the control group. There was diminished comfort felt at six weeks by the control group in each of the eight significant questions on the PCCS. The treatment group, however, did not experience this decrease. This may be explained by providing continuous feedback for the treatment group that eased their transition in the internship year and allowed them to build confidence in their clinical skills, which may have assisted them in assimilating new information faster. On the other hand, such resource-intensive intervention was done on a small sample size and should have been done on a larger sample. Further research is needed to support the implementation of this DO/FF approach in internship and residency programs.

Geel et al. [23] searched for the effects of practice-instruction order and the ratio of normal images on the outcomes of interpreting medical images. They found the deductive method was better than the inductive approach in judging normal from abnormal CXR, and one weakness was considering third-year students as novices in image interpretation when they could have acquired some knowledge of chest pathology during their previous medical years. This could have been enough for them to benefit from the deductive approach, whereas the inductive method is recommended for completely novice and less-experienced students such as those in their first year. The authors recommended the replication of their study on novice-level students. Additional findings were that the ratio of normal to abnormal CXRs affected the criteria by which students categorized normal and abnormal CXRs. This finding was in line with their hypothesis based on the findings of Pusic et al. [35] regarding the effect of changing the proportion of normal images in a study on residents, which the authors replicated on novice medical students revealing that a short training session could have affected this criterion. Therefore, a mismatch in the percentage of abnormalities in training versus the medical images in the clinical practice can easily result in suboptimal criterion training, but it is unlikely that it can lead to an increase in false-negative interpretations. So, the percentage and prevalence of diseases to be considered during the training development should be considered.

This review recognizes several areas for improvement, which can be addressed in prospective studies. First, the small number of the included studies could have affected its results; nonetheless, it provides invaluable insights. Second, the small sample size of some studies might not reflect the actual impact of the interventions. Therefore, remote/online studies should be explored further as they permit widespread adoption and testing of the tools. Third, with the tools above, more interventions should be done on a larger scale.

## Conclusions

In conclusion, this systematic review explored the literature for competency and the different methods to improve medical students’ and interns’ competency in interpreting CXR. Such a topic was not thoroughly explored, and very few researchers tackled the methods for enhancing outcomes. This review identified several findings that may help in the context of teaching and preparing curricula for students, such as the importance of providing clinical history with the imaging; the use of inductive methods of teaching CXR in early years could have promising results, taking in consideration the prevalence of diseases to adjust the abnormal to normal images when preparing an undergraduate curriculum; the remarkable impact of SAFMEDS on medical students' ability to identify CXR findings and the use of online and e-learning methods have shown promising results due to their availability, affordability, and practicality, especially after the COVID-19 pandemic experience. We recommend that more studies be done to shed light on further approaches to enhance the educational outcomes and capabilities of medical students and interns to prepare them to be safe and competent physicians.
